# Molecular Characterization of HBV Strains Circulating among the Treatment-Naive HIV/HBV Co-Infected Patients of Eastern India

**DOI:** 10.1371/journal.pone.0090432

**Published:** 2014-02-28

**Authors:** Debraj Saha, Ananya Pal, Avik Biswas, Rajesh Panigrahi, Neelakshi Sarkar, Dipanwita Das, Jayeeta Sarkar, Subhasish Kamal Guha, Bibhuti Saha, Sekhar Chakrabarti, Runu Chakravarty

**Affiliations:** 1 ICMR Virus Unit, Kolkata, ID & BG Hospital Campus, Kolkata, West Bengal, India; 2 Calcutta School of Tropical Medicine, Kolkata, West Bengal, India; 3 Department of Pathology and Laboratory Medicine, Tulane University School of Medicine, New Orleans, Louisiana, United States of America; 4 National Institute of Cholera and Enteric Diseases, Kolkata, West Bengal, India; Saint Louis University, United States of America

## Abstract

Previously we reported that the exposure to hepatitis B virus (HBV) infection serves as a major threat among the treatment naive HIV infected population of eastern India. Hence, molecular characterization of these strains is of utmost importance in order to identify clinically significant HBV mutations. A total of 85 treatment naive HIV/HBV co-infected participants were included of whom the complete basal core promoter/precore region, the core and the whole envelope gene could be successfully sequenced for 59, 57 and 39 isolates respectively. Following phylogenetic analysis, it was found that HBV/D was the predominant genotype with HBV/D2 (38.5%) being the most prevalent subgenotype followed by HBV/A1. The major mutations affecting HBeAg expression includes the A1762T/G1764A (13.6%), G1896A (22%) and G1862T mutation (33.9%) which was predominantly associated with HBV/A1. Moreover, the prevalence of G1896A was considerably high among the HBeAg negative HIV/HBV co-infected subjects compared to HBV mono-infection. The main amino acid substitutions within the MHC class II restricted T-cell epitope of HBcAg includes the T12S (15.8%) and T67N (12.3%) mutation and the V27I (10.5%) mutation in the MHC class I restricted T-cell epitope. PreS1/S2 deletion was detected in 3 isolates with all harboring the BCP double mutation. Furthermore, the frequently occurring mutations in the major hydrophilic loop of the S gene include the T125M, A128V and M133I/L. Therefore, this study is the first from India to report useful information on the molecular heterogeneity of the HBV strains circulating among the treatment naive HIV/HBV co-infected population and is thus clinically relevant.

## Introduction

Hepatitis B virus (HBV) infection remains a global health problem in spite of an effective vaccine, with approximately 400 million people being chronically infected with HBV worldwide [1]. HBV, the archetype member of the *Hepadnaviridae* family, is a partially double stranded DNA virus of approximately 3.2 kb in genome length with four overlapping open reading frames encoding the polymerase (P), nucleocapsid (C), envelope (S), and X protein [2].

Co-infection with HBV is frequent among the human immunodeficiency virus (HIV) infected individuals due to their similar transmission routes [3]. Presence of HIV might alter the natural history of HBV infection resulting in high serum HBV DNA levels, lower HBeAg to anti-HBe conversion rates and increased risk of liver cirrhosis and hepatocellular carcinoma (HCC) [4], [5], [6]. HBV replicates by means of an error prone reverse transcriptase phenomenon which leads to different mutations along the HBV genome which might have detrimental effect on the natural course of the infection [7]. For example, the classical A1762T/G1764A double mutation within the basal core promoter (nt 1742–1849 from *EcoR*I site) and the G1896A mutation in the precore (PC) (nt 1814–1900 from *EcoR*I site) region are often reported to be associated with advanced liver diseases including liver cirrhosis and HCC in HBV mono-infection [8], [9], [10], [11]. Similarly, the presence of Pre-S1/S2 deletion have also been reported to be associated with progressive liver disease [12] and increased risk for HCC [13] in HBV mono-infection. Moreover, the frequency of PreS2 deletion was previously found to be higher among the HIV/HBV co-infected population [14].

Reports on HIV/HBV co-infection from India are rare. In our previous study we showed that the rate of HBV infection was considerably high among the HIV infected population of eastern India with majority of them being associated with high HBV DNA levels [15]. However, molecular characterization of the HBV strains circulating among the HIV infected cohort remains to be analyzed. Therefore, the present study aims to characterize the different HBV mutations found among the HIV/HBV co-infected population of eastern India which might provide newer insights into the possible reasons for the proficiency of the infection.

## Methods

### Ethics Statement

The present study was approved by the Institutional Ethical Committee of National Institute of Cholera and Enteric Diseases (ICMR) with written informed consent being obtained from all the study participants in their native language.

### Study Subjects

Of the 119 HIV/HBV co-infected patients who participated in our previous study [15], sufficient sample was available from 85 isolates for further analysis at the molecular level and they were thus included in this present study. Data regarding age, sex, possible modes of transmission and different HBV sero-markers were previously abstracted from all the participants. Furthermore, of all the HIV/HBV co-infected participants included for this present study, 12 patients (12/85, 14.1%) had developed acquired immunodeficiency syndrome (AIDS).

### DNA Isolation, PCR Amplification and Sequencing of Different HBV Region

DNA was extracted from the HIV/HBV co-infected subjects using QIAamp DNA Blood Kit (Qiagen, Hilden, Germany) according to manufacturer’s protocol. The HBV genotyping was initially done by a highly sensitive polymerase chain reaction-restriction fragment length polymorphism (PCR-RFLP) method, as previously described [16]. The complete basal core promoter/precore (BCP/PC) (nt 1742–1900 from *EcoR*I site) region was amplified by nested PCR with first round primers ep1-1 (sense)/ep1-2 (antisense) and second round primers ep2-1 (sense)/ep2-2 (antisense) whereas the complete core gene (nt 1901–2452 from *EcoR*I site) was amplified using the primers HB7F (sense)/HBAS6 (antisense) for the first round and CB3 (sense)/HBAS5 (antisense) for the second round.

Similarly, the complete PreS1/S2 and small surface (S) gene was amplified by heminested PCR in two different fragments. The first fragment includes the whole PreS1/S2 region which was amplified as described earlier [17] while the second fragment, spanning the entire S gene, was amplified using primers HB1F (sense)/HS4R (antisense) for the first round and B2 (sense)/HS4R (antisense) for the second round. The above reactions were performed using Promega Taq DNA polymerase (Promega, Madison, WI) and all the reagent concentrations were maintained as per the earlier methods [16]. Details of all the primers and their respective thermal profiles are presented in Table 1.

**Table 1:** List of primers used to amplify the different regions of the HBV genome and their respective thermal profile are presented here.

HBV region/Round	Primer Name (Position)	Primer Sequence (5′-3′)		Thermal Profile
Basal core promoter/Precore region				
First	Ep1-1 (1606–1625)	GCATGGAGACCACCGTGAAC		94°C (30 s), 55°C (30 s), 72°C (45 s)/35 cycle
	Ep1-2 (1974–1955)	GGAAAGAAGTCAGAAGGCAA		
Second	Ep2-1 (1653–1672)	CATAAGAGGACTCTTGGACT		94°C (30 s), 55°C (30 s), 72°C (45 s)/30 cycle
	Ep2-2 (1959–1940)	GGCAAAAAAGAGAGTAACTC		
Core gene				
First	HB7F (1611–1630)	GAGACCACCGTGAACGCCCA		94°C (45 s), 55°C (45 s), 72°C (60 s)/40 cycle
	HBAS6 (2993–2971)	GGGTTGAAGTCCCAATCTGGATT		
Second	CB3 (1824–1843)	TTCACCTCTGCCTAATCATC		94°C (45 s), 55°C (45 s), 72°C (60 s)/35 cycle
	HBAS5 (2662–2643)	GGATAGAACCTAGCAGGCAT		
PreS1/S2				
First	HB7F (1611–1630)	GAGACCACCGTGAACGCCCA	94°C (60 s), 55°C (60 s), 72°C (60 s)/45 cycle	
	HB12R (256–237)	CGAGTCTAGACTCTGTGGTA		
Second	HBS6 (2814–2834)	GGGTCACCATATTCTTGGGAA	94°C (60 s), 55°C (60 s), 72°C (60 s)/40 cycle	
	HB12R (256–237)	CGAGTCTAGACTCTGTGGTA		
S gene				
First	HB1F (18–39)	AAGCTCTGCTAGATCCCAGAGT	94°C (45 s), 55°C (45 s), 72°C (60 s)/40 cycle	
	HS4R (989–970)	CATACTTTCCAATCAATAGG		
Second	B2 (65–84)	GGCTC (AC) AGTTC (AC) GGAACAGT	94°C (45 s), 55°C (45 s), 72°C (60 s)/35 cycle	
	HS4R (989–970)	CATACTTTCCAATCAATAGG		

After PCR amplification the amplicons were directly sequenced in both directions using their respective second round primers, prism Big Dye kit and ABI 3130xl Genetic Analyzer (Applied Biosystems, Foster City, USA). All the sequences were aligned and corrected using BioEdit v7.1.3.0 [18]. The BCP/PC and the core sequences were joined via the overlapping regions to acquire a 711 bp (HBV/D and HBV/C) and a 717 bp (HBV/A) sequence. Similarly, the complete S region was amplified in two fragments which were joined to obtain a maximum sequence length of 1170 bp for HBV/D and 1203 bp for HBV/A & HBV/C initiating from preS1 start codon. The accession numbers for the HBV BCP/PC/core gene sequenced in this study are KF798259 - KF798315; for the complete small S gene are KF798220 - KF798258 and the two additional isolates whose BCP/PC region could be sequenced are KF798316 - KF798317.

### Phylogenetic Analysis

HBV genotypes and subgenotypes for the isolates were determined phylogenetically using MEGA 5 [19]. The phylogenetic tree was constructed using the neighbor-joining method [20] and the reliability of the tree was evaluated by means of bootstrap analysis with 1,000 replicates. Pairwise evolutionary distances were computed using the Kimura two-parameter model [21].

### Statistical Analysis

Data entries, determining the median of different parameters and other preliminary calculations were done using Microsoft Excel. Mann-Whitney test was used for comparing the medians of continuous variables using Graphpad Prism (version 4.0.3). Chi-square or Fischer’s exact test was used for comparing categorical data using StatCalc (EpiInfo version 6.0, Centers for Disease Control and Prevention and World Health Organization, Geneva, Switzerland). In all the analyses, p-values <0.05 were considered as statistically significant.

## Results

Among the 85 isolates included for this study, the complete BCP/PC region could be successfully analyzed for 59 treatment naive HIV/HBV co-infected subjects. On the other hand, the entire core gene could only be successfully sequenced for 57 isolates. Among the patients whose complete BCP/PC region was sequenced, the complete envelope gene (PreS1, PreS2 and S) of 39 HBV isolates could be amplified.

### HBV Genotyping and Baseline Characteristics of the Study Subjects

Among the above 59 study subjects, 53 (89.8%) were males and 6 (10.2%) were females. HBV genotyping, done by PCR-RFLP method, showed that 34 of the 59 HIV/HBV co-infected patients belonged to HBV/D (57.6%). The major baseline characteristics of these study participants are summarized in Table 2.

**Table 2:** Baseline characteristics of all the participating HIV/HBV co-infected individuals belonging to different HBV genotypes.

Variable	Total HIV/HBV patients	Genotype		
		HBV/A	HBV/C	HBV/D
Patients, n (%)	59	19 (32.2)	6 (10.2)	34 (57.6)
Age, years, median (IQR)	35 (21–60)	35 (21–60)	33.5 (26–50)	34.5 (32–54)
Male sex, n (%)	53 (89.8)	17 (89.5)	6 (100)	30 (88.2)
CD4+ T-cell count, cells/mm^3^, median (IQR)	198 (26–838)	144 (61–838)	319.5 (186–629)	194 (26–414)
ALT, U/L, median (IQR)	50 (15–406)	54 (15–186)	52 (17–406)	45 (22–182)
HBeAg positive, n (%)	46 (78)	15 (79)	4 (66.7)	27 (79.4)
HBV DNA, log_10_IU/ml, median (IQR)	5.7 (2–7.65)	5.7 (2–7.64)	4.9 (4.62–5.96)	5.8 (4.37–7.65)

The median CD4+ T-cell count for HIV/HBV co-infected patients infected with either HBV/A (144 cells/mm^3^, inter-quartile range [IQR] 61–838 cells/mm^3^) or HBV/D (194 cells/mm^3^, IQR 26–414 cells/mm^3^) was considerably lower compared to subjects infected with HBV/C (319.5 cells/mm^3^, IQR 186–629 cells/mm^3^). However, this difference was statistically not significant (HBV/A vs. HBV/C; p = 0.14 & HBV/D vs. HBV/C; p = 0.09). Moreover, there was no significant difference among subjects infected with either of the HBV genotypes with respect to ALT levels or the HBV DNA load.

### Phylogenetic Analysis and Genetic Diversity

**Figure 1:**
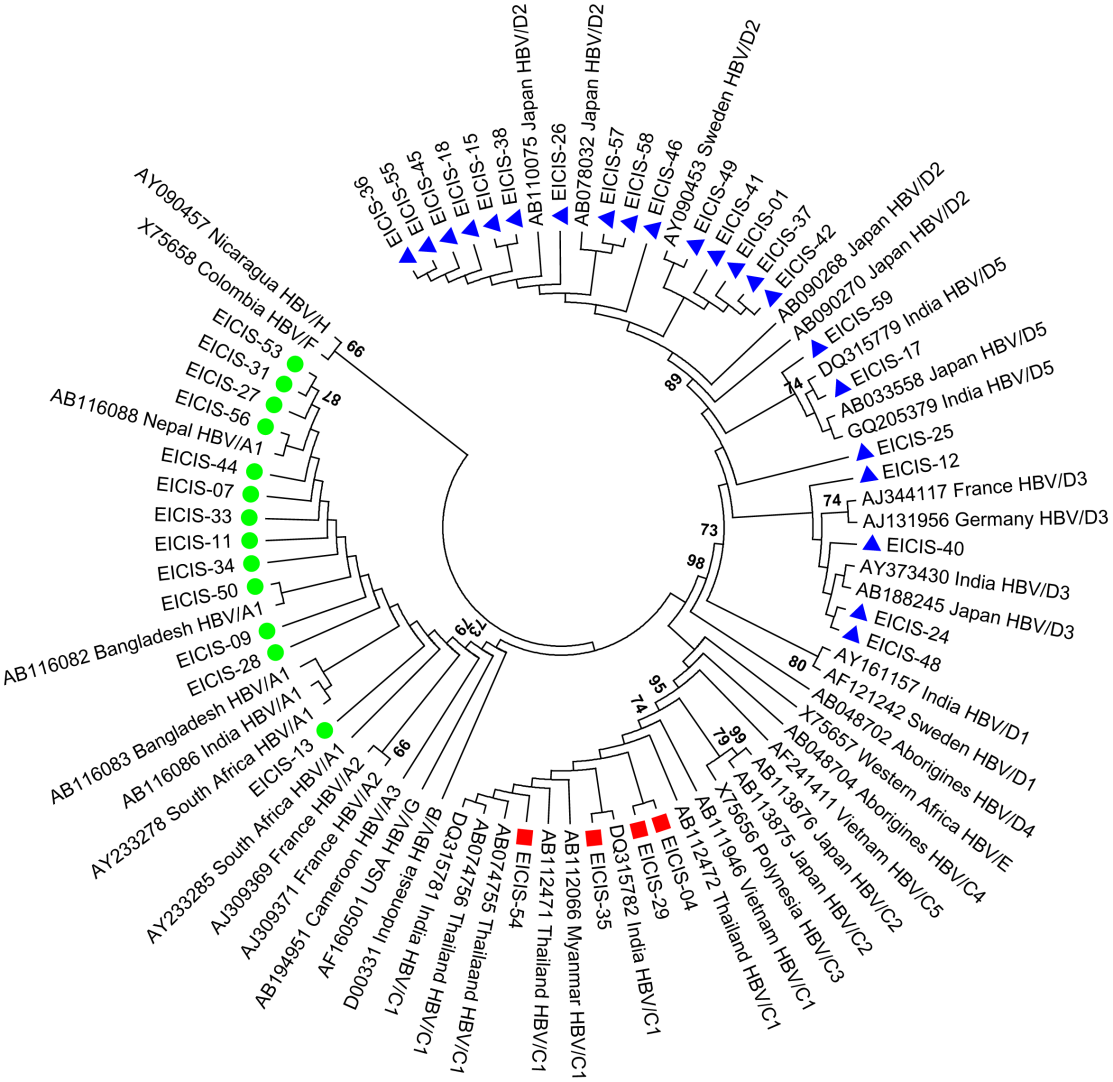
Phylogenetic analysis of the HBV isolates circulating among the HIV/HBV co-infected population of eastern India. The phylogenetic tree was constructed based on the complete small S region (nucleotide 155–835 from *EcoR*I site) of the HBV genome using the neighbor-joining method and bootstrap value of 1000 replicates. The 39 isolates (denoted by EICIS) were analyzed with respect to 42 reference sequences retrieved from the GenBank which are designated by their respective accession numbers along with their HBV genotypes/subgenotypes and country of origin. • represents isolates belonging to HBV/A, ▪ represents HBV/C whereas ▴ represents HBV/D.

Phylogenetic analysis was done based on the complete small S gene (nt 155–835 from *EcoR*I site) of the HBV genome. The 39 representative isolates were analyzed along with 42 reference sequences belonging to different HBV genotypes/subgenotypes retrieved from the GenBank [Figure 1]. Such analysis also showed that HBV/D was the predominant genotype which was further distributed among three subgenotypes HBV/D2, HBV/D3 & HBV/D5 with HBV/D2 (15/39, 38.5%) being the most prevalent subgenotype circulating among the HIV/HBV co-infected patients of eastern India.

**Figure 2:**
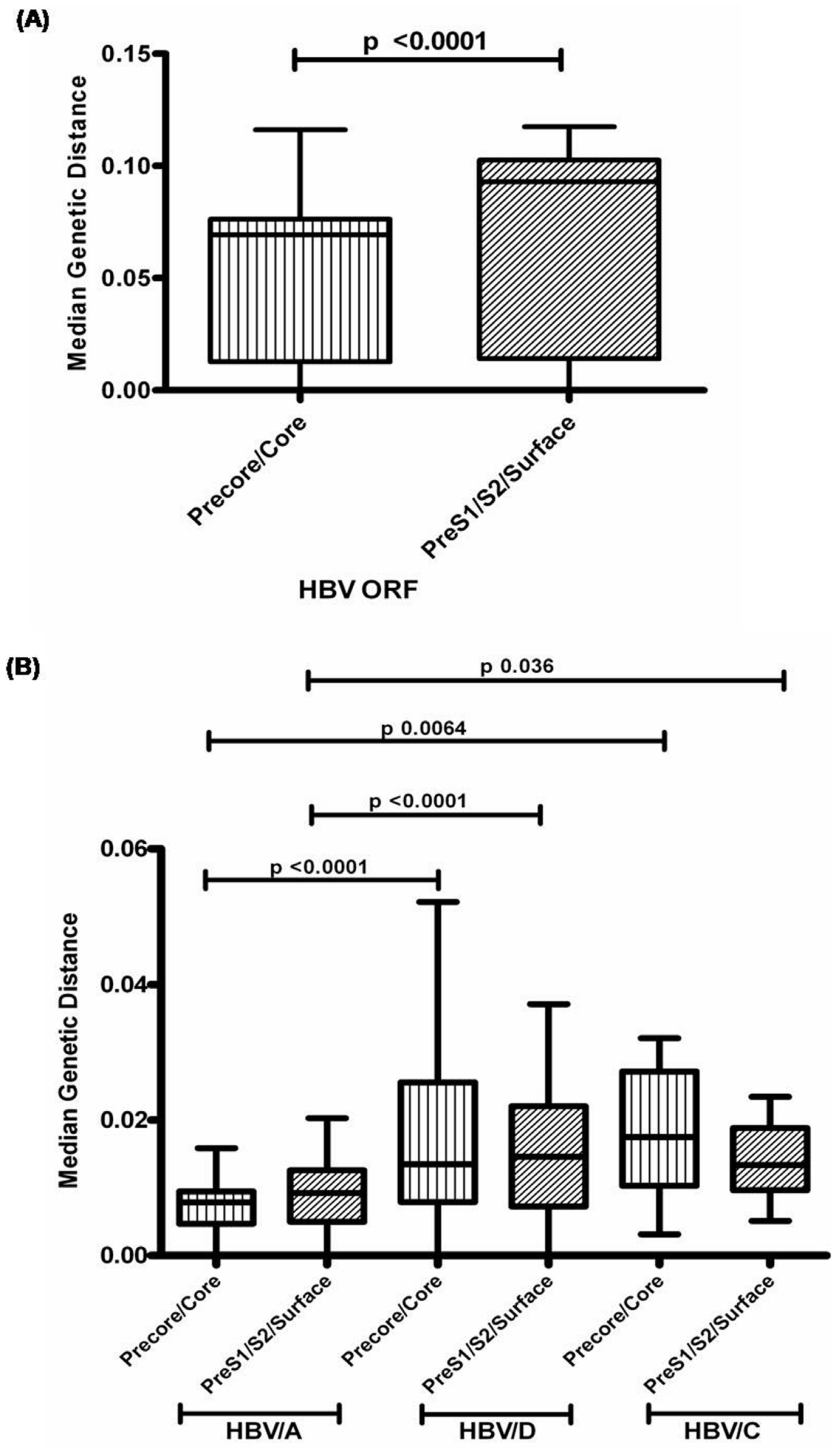
Inter-patient genetic distances for different HBV genes and its divergence among different HBV genotypes. (A) The median genetic distance was significantly higher for the HBV PreS1/S2/Surface region compared to the precore/core regions. (B) The median genetic distance was significantly lower in case of HBV/A compared to HBV/D and HBV/C for both the HBV open reading frames (ORF), thus indicating the low genetic diversity of HBV/A.

The overall inter-patient genetic distance was calculated for different regions of HBV genome and it was found that the median genetic distance was significantly higher for the HBV PreS1/S2/Surface region compared to the Precore/core gene (p =  <0.0001) [Figure 2A]. Moreover, it was found that the median genetic distance was significantly lower for both the PreS1/S2/Surface and the Precore/core region in case of HBV/A strains compared to HBV/D and HBV/C [Figure 2B].

### Mutational Analysis of the BCP and Precore Region

**Figure 3:**
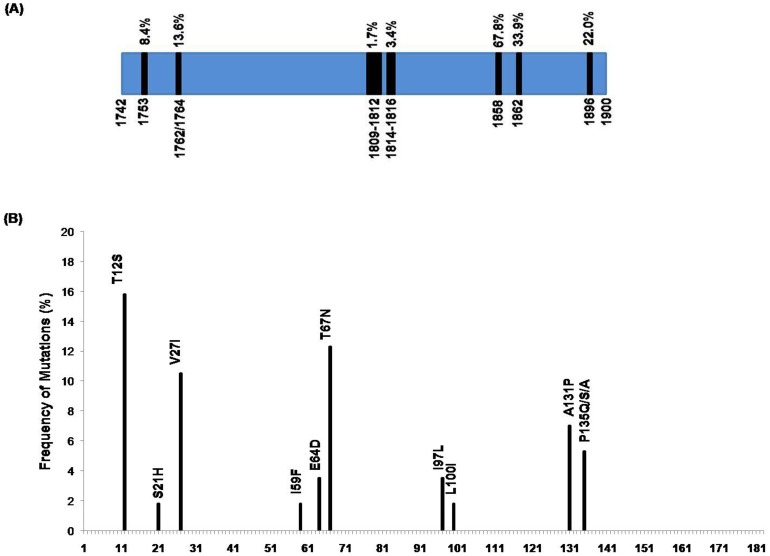
Mutations in the Basal Core Promoter/PreCore and the Core regions of the HBV Genome. (A) The frequency of the major mutations found in the BCP/precore region of the HBV strains circulating among the treatment naive HIV infected cohort of eastern India are presented here. The frequency of the G1896A precore mutation was higher compared to the BCP double mutations in our settings with majority of the strains harboring the C1858T mutation. (B) The frequencies of the amino acid mutations found in the immune-active regions of the core gene are shown. The major mutations in the MHC class I restricted (amino acids 18–27, 88–96, 130–140, 141–151) and MHC class II-restricted (amino acids 1–20, 50–69, 81–105, 117–131, 141–165) T-cell epitopes of core antigen includes the V27I and T12S respectively.

Of the 59 isolates whose complete BCP/PC region was sequenced, 46 were HBeAg positive and 13 were HBeAg negative. The major mutations found in our study population are summarized in Figure 3A. A total of 8 isolates (8/59, 13.6%) harbored the BCP (A1762T/G1764A) double mutations. In 4 cases it occurred together with the T1753C mutation whereas in 5 cases the BCP mutations were accompanied by the G1896A precore mutation. Similarly, the precore stop codon mutation (G1896A) occurred in 13 isolates (13/59, 22%) with all harboring the C1858T mutation and in 4 cases it occurred along with G1899A mutation. The frequency of both BCP and precore mutations was significantly higher for the HBeAg negative HIV/HBV co-infected subjects compared to HBeAg positive subjects (38.5% vs. 6.5%; p = 0.009 & 61.5% vs. 10.9%; p = <0.0001 respectively).The precore stop codon mutation was predominantly found in HBV/D (10/34, 29.4%) and in majority of the HBV/C strains while none of the HBV/A isolates had this mutation. On the other hand, the BCP double mutation was common among the HBV/A and HBV/C strains compared to HBV/D.

The Kozak sequence mutation (nt 1809–1812 from *EcoR*I site) was found in only one HBV/A isolate harboring the A1811C mutation. Similarly, the A1814C precore start codon mutation was found in two isolates. Furthermore, the G1862T mutation was found in 33.9% (20/59) of the study subjects. Strikingly, all the HBV/A isolates harbored this mutation.

### Mutations in the Core Gene

Several amino acid substitutions were found within the immune-active region of the HBV core gene including the T12S (9/57, 15.8%) and T67N (7/57, 12.3%) mutation in the MHC class II restricted T-cell epitope of HBV core protein and the V27I (6/57, 10.5%) mutation in the MHC class I restricted T-cell epitope. Majority of these mutations were associated with HBV/C strains. Other mutations found within the immunologically-active region of the core protein are presented in Figure 3B. Furthermore, the −1 G frameshift mutation was detected in one of the isolate belonging to HBV/A.

### Analysis of the Complete S Gene

**Figure 4:**
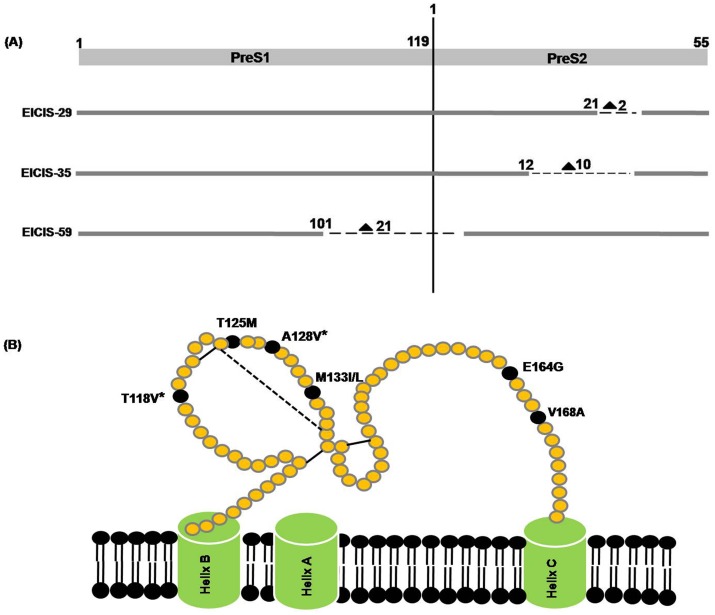
Schematic representation of the PreS deletions and the mutations in the small surface gene of the HBV strains isolated from the HIV/HBV co-infected patients of eastern India. (A) The PreS deletions are indicated by dashed lines (–). The numbers ahead of each deletion indicates the starting point of the respective amino acid deletions whereas the numbers besides the triangle (▴) indicates the length of each deletion observed in the co-infected individuals. (B) The major mutations occurring frequently within the small surface gene of HBV are shown here. The substitutions marked with a star (*) are mainly contributed due to the presence of the HBV/D2 sequences.

Among the 39 HIV/HBV co-infected subjects, whose complete envelope gene could be successfully sequenced, the PreS1/S2 deletion was detected in 3 isolates (3/39, 7.7%) [Figure 4A]. Two isolates, *EICIS-29* & *EICIS-35,* had a deletion of 2 and 10 amino acids respectively in the PreS2 domain whereas *EICIS-59* had a large deletion of 21 amino acids in the PreS1 region of the envelope gene. Interestingly, all the subjects with PreS1 or PreS2 deletion had A1762T/G1764A mutation in their BCP region whereas the G1896A PC mutation was found in two of the isolates. Very few amino acid substitutions were found within the hepatocyte binding site including the G27S and G35R mutation in only one HBV/C isolate and A39S/R mutation in 3 HBV/D isolates.

The mutations which occurred more frequently within the small S gene are shown in Figure 4B. In addition, other mutations such as Y100D, Q101R/H, M103V/I, D144E and G145S was also found within the major hydrophilic loop (MHL) of the S gene. Moreover, 93.3% of the HBV/D2 strains had T118V (14/15) whereas all the HBV/D2 isolates were associated with A128V. Additionally, two premature stop codon mutations, the C69* and the W182* mutation, were observed outside the MHL region in two isolates.

## Discussion

Despite India being intermediately endemic for both HIV and HBV, there are very few reports on HIV/HBV co-infection from India and even scarce from eastern India. In our previous study, we showed that the exposure to HBV co-infection was predominant among the ART naive HIV infected population of eastern India and thus it serves as a major threat in this population [15]. However, the molecular characterization of these HBV strains remained to be done. Therefore, the present study primarily emphasizes on the genetic variability of different HBV regions and to best of our knowledge, is the first in India to evaluate the prevalence of different HBV mutations among the treatment naive HIV/HBV co-infected population. Several clinically significant HBV mutations were observed in our settings including the BCP double mutations, PreS deletions and the G1896A precore mutation, which was predominant among the HBeAg negative HIV/HBV co-infected patients who were treatment naive.

In our recent report, we showed that HBV/D was the predominant genotype circulating among the ART naive HIV/HBV co-infected population of eastern India followed by HBV/A and HBV/C [15]. In the present study, we showed that HBV/D2 (15/39, 38.5%) was the major subgenotype prevailing among the HIV/HBV co- infected patients of eastern India whereas the most prevalent non-HBV/D genotype was HBV/A1 (13/39, 33.3%). This was in accordance with our previous study which also showed similar results [22]. Moreover, the genetic diversity of HBV/D or HBV/C was significantly higher compared to HBV/A which was reflected by high inter-patient genetic distances for different HBV open reading frames (ORF) including the PreS1/S2/Surface and the precore/core region [Figure 4B]. These findings were similar to earlier studies on HBV mono-infection which also suggested that HBV/D exhibits high degree of molecular heterogeneity [23], [24] and has an estimated substitution rate higher than that of HBV/A [25].

The core promoter (nt 1613–1849 from *EcoR*I site) region of HBV is of great importance in HBV replication. It consists of two regulatory regions: the upper regulatory region and the basal core promoter [26]. The BCP region is composed of 4 TA rich sequences which play a pivotal role during the transcription process [10] and are considered as mutational “hot spots” [26]. The complete analysis of the BCP and the precore/core region in this study showed that 13.6% of the isolates harbored the BCP double mutation whereas about 22% of them had the G1896A precore mutation. However, the prevalence of the A1762T/G1764A mutations were relatively lower in case of the HIV/HBV co-infected patients, as our previous study on HBV mono-infection from eastern India showed that about 61% of the subjects harbored this double mutation [10]. In a study performed by Audsley et al, it was also shown that the frequency the BCP double mutation was considerably high in case of HBV mono-infection compared to HIV/HBV co-infection [14]. Possibly, the presence of more HBeAg positive participants in our study might have led to the overall decrease in the frequency of the BCP mutations which are more often associated with HBeAg negative status. On the other hand, the prevalence of both BCP double mutation and G1896A were considerably low among the HBeAg positive HIV/HBV co-infected patients which was in accordance with our previous reports [27]. Interestingly, in contrast to our earlier reports which showed that 18–24% of the HBeAg negative HBV mono-infected patients harbored the G1896A mutation [27], [10], the prevalence of G1896A precore mutation was considerably high (61.5%) among the HBeAg negative HIV/HBV co-infected subjects from eastern India. However, presence of any distinct BCP or Precore mutations distinguishing between HBeAg positive and HBeAg negative HIV/HBV co-infected patients was not observed in our study population.

Our findings also revealed that the A1762T/G1764A mutations were more common among HIV/HBV co-infected subjects who were infected with HBV/A or HBV/C whereas the G1896A precore mutation was more associated with HBV/D which was in concordance with earlier studies on HBV mono-infection from this part of India [10], [28]. Several reports suggest that the BCP double mutations, HBV/A and HBV/C are considered as a major risk factors for severe liver disease [29], [30]. In the present study, 50% of the subjects infected with HBV/C harbored the A1762T/G1764A mutations, thus increasing the chances of advanced liver disease among the HIV/HBV co-infected cohort. On the other hand, the clinical relevance of G1896A mutation is ambiguous, with some reporting its association with severe forms of chronic liver disease [31] while other studies suggest no such relevant association [8], [32]. As per previous reports from India, the presence of G1896A might lead to inactivation of liver diseases [33]. Therefore, since we found that the frequency of G1896A mutation was noticeably high among the HBeAg negative HIV/HBV co-infected individuals, further studies are needed to monitor the clinical outcome of these patients in future and examine whether G1896A has any clinical significance in this cohort.

The Kozak sequence mutation which suppresses the translation of HBeAg [34] and the precore start codon mutation which completely terminates the expression of HBeAg [35] was also found in our study among the HBV/A strains but at a lower frequency. Beside these mutations, a common missense mutation at nucleotide 1862 (G to T) was also observed in about 34% of the isolates. The G1862T mutation is known for affecting the expression of HBeAg at the post-translational level [36]. It converts the amino acid residue valine at codon 17 of the precore peptide to phenylalanine, which being a bulkier residue interferes with signal peptidase cleavage [37]. Moreover, all the HBV/A isolates of this present study were associated with the G1862T, irrespective of HBeAg status. A previous study also demonstrated that G1862T mutation was appreciably related to HBV/A1 rather than HBV/A2 [38]. Such significant association between this mutation and HBV/A was earlier reported from our lab among the HBV mono-infected patients from eastern India, which supports the idea that this mutation might be a part of the natural variability of HBV/A1 [39].

Additional random mutations were observed in the immune-active regions of the core gene which consists of different B-cell and T-cell epitopes and is thus vulnerable to different mutations during the course of infection [40]. The major mutations include the T12S, V27I and T67N which were predominant among the HBV/C strains. Moreover, the frequency of the mutations in the MHC-II restricted T-cell epitope was higher compared to MHC-I restricted T-cell epitope, which coincide with other recent findings [41], [42]. A recent study showed that HBV/C related P5H, I97F/L and L100I mutations were significantly associated with HCC [42]. However, the frequency of these mutations was markedly low in our study population. A possible reason might be due to the fact that our cohort comprised of HIV infected individuals and are therefore associated with lower immune selection pressure.

A study on HIV/HBV co-infection had earlier reported the presence of an unusual mutation (−1 G frameshift mutation) predominantly found among this cohort, especially among those infected with HBV/A strains [14]. This mutant, created by the deletion of a guanosine residue from the guanine-rich homopolymer sequence at nts 2,085–2,090 (from *Eco*RI site) in the HBV core gene, results in the formation of a truncated precore or core protein and was more prevalent among patients from USA and Australia [43]. Furthermore, this mutant was also reported in a Japanese patient who was infected with both HIV and HBV [44]. In vitro studies have revealed that the truncated proteins are more often intracellularly retained in the endoplasmic reticulum and the Golgi apparatus [45]. In this study, we found that the frequency of this mutation was very low in our study population as only one HBV/A isolate harbored this unique mutation. However, none of the previous studies from India had reported the presence of this mutation both in case of HIV/HBV co-infection and HBV mono-infection.

Several studies on HBV mono-infection had previously reported the presence of PreS1/S2 deletion [46], [47] which has severe clinical significance. In sub-saharan Africa, it had been reported to be associated with increased risk of hepatocarcinogenesis [48]. Moreover, several other reports have shown that the deletion mutants often accumulate within the endoplasmic reticulum (ER) of hepatocyte and cause ER stress, which might be a possible cause for development of HCC [49]. In our study, we found that the frequency of PreS deletion among the HIV/HBV co-infected population was only 7.7%, which was much lower compared to its prevalence in case of HBV mono-infection (20% PreS1 and 24% PreS2 deletion) from eastern India [17]. This was in contrast to another study where they showed that the frequency of PreS2 deletions were significantly high in HIV/HBV co-infection rather than HBV mono-infection [14]. Notably, it was seen that all the isolates which harbored the PreS1/S2 deletion were accompanied by the BCP A1762T/G1764A double mutations. Similar association was previously noticed in case of HIV/HBV co-infection and also in HBV mono-infected patients with advanced liver diseases [14], [50]. Additionally, the F141L mutation in the PreS region was not found in any of the HIV/HBV co-infected individuals, although this mutation was found to be significant among the liver cirrhosis patients compared to the chronic HBV cases with HBV mono-infection [17].

The current study also focused on the molecular heterogeneity of the small surface gene of the HBV strains circulating among the treatment naive HIV/HBV co-infected population of eastern India. Several amino acid substitutions within the major hydrophilic loop were observed in our study population which included Y100D, Q101R/H, M103V/I, T125M, A128V, M133L, D144E and G145S. The presence of T118V and A128V were found significantly in HBV/D2, which concurs with our previous study [51] and hence are considered as signature residues of this subgenotype. Additional mutation outside the MHL region was also observed including the E164G mutation, which was earlier reported by another study on chronic HBV carriers [52]. However, its implication as a vaccine escape mutant is still unclear. Notably, two premature stop codon mutations, one at codon 69 and another at codon 182, were found in the surface gene of the HBV strains. The former results in a truncated surface protein and was previously reported among the HIV/HBV co-infected patients of eastern India [22]. The W182* mutation had been previously reported to have severe clinical implications. A study involving Korean patients showed that this stop codon mutation was frequently found in HBV/C and is associated with progressive form of liver disease particularly with HCC [53]. In addition, a recent genome wide study showed the association between chronic HBV infection and HLA-locus in a Japanese population. It showed that variations in antigen-binding sites of HLA-DP and HLA-DQ might result in persistent HBV infection [54]. However, similar reports on HIV/HBV co-infection are lacking and needs further study to identify the different host genetic factors associated with HBV infection among the HIV infected patients.

In conclusion, our study mainly highlights the genetic variability of the different HBV genes among the treatment naive HIV/HBV co-infected population of eastern India. To best of our knowledge, it is the first study of its kind from India to report the prevalence of different HBV mutations in the HIV infected cohort. In this study, it was observed in accordance to our previous report, that HBV/D2 was the predominant subgenotype circulating among the HIV/HBV co-infected population followed by HBV/A1. Furthermore, the frequency of the A1762T/G1764A mutations was comparatively lower than its frequency among HBV mono-infected cases from this part of the country with majority of the HBV/C strains being associated with this mutation. However, the prevalence of G1896A was markedly high among the HBeAg negative HIV/HBV co-infected patients and therefore, needs further follow-up to assess whether this mutation is associated with any severe clinical implications in this cohort. Finally, this study for the first time also reports the presence of the −1 G frameshift deletion and the W182* mutations among the Indian population but with a lower frequency. Therefore, the current study provides valuable information on the molecular heterogeneity of the HBV strains circulating among the treatment naive HIV/HBV co-infected population of eastern India and might elucidate the molecular basis for the proficiency of the HBV infection in this cohort.

## References

[1] Soriano V, Barreiro P, Martin-Carbonero L, Castellares C, Ruiz-Sancho A, et al (2007) Treatment of Chronic Hepatitis B or C in HIV-Infected Patients with Dual Viral Hepatitis. J Infect Dis 195(8): 1181–1183. 17357055 10.1086/512679

[2] Seeger C, Mason WS (2000) Hepatitis B virus biology. Microbiol Mol Biol Rev 64(1): 51–68. 10704474 10.1128/mmbr.64.1.51-68.2000 PMC98986

[3] Sellier P, Schnepf N, Jarrin I, Mazeron MC, Simoneau G, et al (2010) Description of liver disease in a cohort of HIV/HBV coinfected patients. J Clin Virol 47(1): 13–17. 19897410 10.1016/j.jcv.2009.10.010

[4] Mathews G, Bhagani S (2003) The epidemiology and natural history of HIV/HBV and HCV co-infections. J HIV Ther 8(4): 77–84. 14671504

[5] Thio CL, Seaberg EC, Skolasky R Jr, Phair J, Visscher B, et al (2002) HIV-1, hepatitis B virus, and risk of liver-related mortality in the Multicenter Cohort Study (MACS). Lancet 360: 1921–1926. 12493258 10.1016/s0140-6736(02)11913-1

[6] Thio CL (2009) Hepatitis B and human immunodeficiency virus co-infection. Hepatology 49(5 Suppl): S138–S145. 10.1002/hep.22883 19399813

[7] Fares MA, Holmes EC (2002) A revised evolutionary history of hepatitis B virus (HBV). J Mol Evol 54(6): 807–814. 12029362 10.1007/s00239-001-0084-z

[8] Kao JH, Chen PJ, Lai MY, Chen DS (2003) Basal core promoter mutations of hepatitis B virus increase the risk of hepatocellular carcinoma in hepatitis B carriers. Gastroenterology 124(2): 327–334. 12557138 10.1053/gast.2003.50053

[9] Liu S, Zhang H, Gu C, Yin J, He Y, et al (2009) Associations between hepatitis B virus mutations and the risk of hepatocellular carcinoma: a meta-analysis. J Natl Cancer Inst 101(15): 1066–1082. 19574418 10.1093/jnci/djp180 PMC2720989

[10] Biswas A, Banerjee A, Chandra PK, Datta S, Panigrahi R, et al (2011) Variations in the functional domain of basal core promoter of Hepatitis B virus among eastern Indian patients with prevalence of genotypes A, C, and D among the same ethnic population. J Med Virol 83(2): 253–260. 21181919 10.1002/jmv.21979

[11] Kitab B, Essaid El Feydi A, Afifi R, Trepo C, Benazzouz M, et al (2012) Variability in the Precore and Core Promoter Regions of HBV Strains in Morocco: Characterization and Impact on Liver Disease Progression. PLoS ONE 7(8): e42891. 22905181 10.1371/journal.pone.0042891 PMC3419231

[12] Sugauchi F, Ohno T, Orito E, Sakugawa H, Ichida T, et al (2003) Influence of hepatitis B virus genotypes on the development of preS deletions and advanced liver disease. J Med Virol 70(4): 537–544. 12794715 10.1002/jmv.10428

[13] Qu L, Kuai X, Liu T, Chen T, Ni Z, et al (2013) Pre-S Deletion and Complex Mutations of Hepatitis B Virus Related to Young Age Hepatocellular Carcinoma in Qidong, China. PLoS ONE 8(3): e59583. 23555717 10.1371/journal.pone.0059583 PMC3610697

[14] Audsley J, Littlejohn M, Yuen L, Sasadeusz J, Ayres A, et al (2010) HBV mutations in untreated HIV–HBV co-infection using genomic length sequencing. Virology 405(2): 539–547. 20655563 10.1016/j.virol.2010.06.038 PMC2935930

[15] Saha D, Pal A, Biswas A, Panigrahi R, Sarkar N, et al (2013) Characterization of Treatment-Naive HIV/HBV Co-Infected Patients Attending ART Clinic of a Tertiary Healthcare Centre in Eastern India. PLoS ONE 8(8): e73613. 24023688 10.1371/journal.pone.0073613 PMC3758335

[16] Biswas A, Chandra PK, Datta S, Panigrahi R, Banerjee A, et al (2009) Frequency and distribution of hepatitis B virus genotypes among eastern Indian voluntary blood donors: Association with precore and basal core promoter mutations. Hepatol Res 39(1): 53–59. 18713275 10.1111/j.1872-034X.2008.00403.x

[17] Biswas A, Panigrahi R, Banerjee A, Pal M, De BK, et al (2012) Differential pattern of pre-S mutations/deletions and its association with hepatitis B virus genotypes in Eastern India. Infect Genet Evol 12(2): 384–391. 22266243 10.1016/j.meegid.2012.01.007

[18] Hall TA (1999) BioEdit: a user-friendly biological sequence alignment editor and analysis program for Windows 95/98/NT. Nucleic Acid Symp Ser 41: 95–98.

[19] Tamura K, Peterson D, Peterson N, Stecher G, Nei M, et al (2011) MEGA5: molecular evolutionary genetics analysis using maximum likelihood, evolutionary distance, and maximum parsimony methods. Mol Biol Evol 28(10): 2731–2739. 21546353 10.1093/molbev/msr121 PMC3203626

[20] Saitou N, Nei M (1987) The neighbor-joining method: a new method for reconstructing phylogenetic trees. Mol Biol Evol 4: 406–425. 3447015 10.1093/oxfordjournals.molbev.a040454

[21] Kimura M (1980) A simple method for estimating evolutionary rates of base substitutions through comparative studies of nucleotide sequences. J Mol Evol 16: 111–120. 7463489 10.1007/BF01731581

[22] Pal A, Panigrahi R, Biswas A, Datta S, Sarkar N, et al (2013) Influence of HIV associated degree of immune suppression on molecular heterogeneity of hepatitis B virus among HIV co-infected patients. Virology 436(1): 134–142. 23228859 10.1016/j.virol.2012.11.003

[23] Zehender G, Ebranati E, Gabanelli E, Shkjezi R, Lai A, et al (2012) Spatial and temporal dynamics of hepatitis B virus D genotype in Europe and the Mediterranean Basin. PLoS ONE 7(5): e37198. 22662136 10.1371/journal.pone.0037198 PMC3360700

[24] Norder H, Couroucé AM, Coursaget P, Echevarria JM, Lee SD, et al (2004) Genetic diversity of hepatitis B virus strains derived worldwide: genotypes, subgenotypes, and HBsAg subtypes. Intervirology 47(6): 289–309. 15564741 10.1159/000080872

[25] Zehender G, Maddalena CD, Giambelli C, Milazzo L, Schiavini M, et al (2008) Different evolutionary rates and epidemic growth of hepatitis B virus genotypes A and D. Virology. 380(1): 84–90. 10.1016/j.virol.2008.07.009 18715605

[26] Kramvis A, Kew MC (1999) The core promoter of hepatitis B virus. J Viral Hepat 6: 415–427. 10607259 10.1046/j.1365-2893.1999.00189.x

[27] Banerjee A, Banerjee S, Chowdhury A, Santra A, Chowdhury S, et al (2005) Nucleic acid sequence analysis of BCP/precore/core region of hepatitis B virus isolated from chronic carriers of the virus from Kolkata, Eastern India: Low frequency of mutation in the precore region. Intervirology 48(6): 389–399. 16024943 10.1159/000086066

[28] Datta S, Biswas A, Chandra PK, Banerjee A, Panigrahi R, et al (2008) Molecular Epidemiology and Clinical Significance of Hepatitis B Virus Genotypes, Core Promoter and Precore Mutations in Eastern India. Intervirology 51: 275–284. 18987483 10.1159/000170902

[29] Kumar A, Kumar SI, Pandey R, Naik S, Aggarwal R (2005) Hepatitis B virus genotype A is more often associated with severe liver disease in northern India than is genotype D. Indian J Gastroenterol. 24: 19–22. 15778521

[30] Chan HL, Hui AY, Wong ML, Tse AM, Hung LC, et al (2004) Genotype C hepatitis B virus infection is associated with an increased risk of hepatocellular carcinoma. Gut 53(10): 1494–1498. 15361502 10.1136/gut.2003.033324 PMC1774221

[31] Tong MJ, Blatt LM, Kao JH, Cheng JT, Corey WG (2007) Basal core promoter T1762/A1764 and precore A1896 gene mutations in hepatitis B surface antigen positive hepatocellular carcinoma: a comparison with chronic carriers. Liver Int 27: 1356–1363. 17900245 10.1111/j.1478-3231.2007.01585.x PMC2229667

[32] Bozdayi AM, Bozkaya H, Turkyilmaz A, Aslan N, Verdi H, et al (1999) Polymorphism of precore region of hepatitis B virus DNA among patients with chronic HBV infection in Turkey. Infection 27: 357–360. 10624597 10.1007/s150100050043

[33] Gandhe SS, Chadha MS, Walimbe AM, Arankalle VA (2003) Hepatitis B virus: prevalence of precore/core promoter mutants in different clinical categories of Indian patients. J Viral Hepat 10: 367–382. 12969189 10.1046/j.1365-2893.2003.00445.x

[34] Ahn SH, Kramvis A, Kawai S, Spangenberg HC, Li J, et al (2003) Sequence variation upstream of precore translation initiation codon reduces hepatitis B virus e antigen production. Gastroenterology 125: 1370–1378. 14598253 10.1016/j.gastro.2003.07.016

[35] Laras A, Koskinas J, Avgidis K, Hadziyannis SJ (1998) Incidence and clinical significance of hepatitis B virus precore gene translation initiation mutations in e antigen-negative patients. J Viral Hepat 5: 241–248. 9751010 10.1046/j.1365-2893.1998.00109.x

[36] Hou J, Lin Y, Waters J, Wang Z, Min J, et al (2002) Detection and significance of a G1862T variant of hepatitis B virus in Chinese patients with fulminant hepatitis. J Gen Virol 83: 2291–2298. 12185284 10.1099/0022-1317-83-9-2291

[37] Nielsen H, Engelbrecht J, Brunak S, von Heijen G (1997) Identification of prokaryotic and eukaryotic signal peptides and prediction of their cleavage sites. Protein Eng 10: 1–6. 10.1093/protein/10.1.1 9051728

[38] Tanaka Y, Hasegawa I, Kato T, Orito E, Hirashima N, et al (2004) A case control study for differences among hepatitis B virus infections of genotypes A (subtypes Aa and Ae) and D. Hepatology. 40: 747–755. 10.1002/hep.20365 15349915

[39] Chandra PK, Banerjee A, Datta S, Chakravarty R (2007) G1862T mutation among Hepatitis B virus infected individuals: Association with viral genotypes and disease outcome in Kolkata, Eastern India. Intervirology 50: 173–180. 17259736 10.1159/000098960

[40] Hur GM, Lee YI, Suh DJ, Lee JH, Lee YI (1996) Gradual accumulation of mutations in precore core region of HBV in patients with chronic active hepatitis: implications of clustering changes in a small region of the HBV core region. J Med Virol 48: 38–46. 8825708 10.1002/(SICI)1096-9071(199601)48:1<38::AID-JMV6>3.0.CO;2-M

[41] Mayaphi SH, Martin DJ, Mphahlele MJ, Blackard JT, Bowyer SM (2013) Variability of the preC/C region of hepatitis B virus genotype A from a South African cohort predominantly infected with HIV. J Med Virol 85(11): 1883–1892. 23925707 10.1002/jmv.23695 PMC4559853

[42] Kim DW, Lee SA, Hwang ES, Kook YH, Kim BJ (2012) Naturally occurring precore/core region mutations of hepatitis B virus genotype C related to hepatocellular carcinoma. PLoS ONE 7(10): e47372. 23071796 10.1371/journal.pone.0047372 PMC3468518

[43] Revill PA, Littlejohn M, Ayres A, Yuen L, Colledge D, et al (2007) Identification of a novel hepatitis B virus precore/core deletion mutant in HIV/hepatitis B virus co-infected individuals. AIDS 21: 1701–1710. 17690567 10.1097/QAD.0b013e32826fb305

[44] Fukushima K, Ueno Y, Inoue J, Wakui Y, Obara N, et al (2008) A case of HIV co-infected with hepatitis B virus precore/core deletion mutant treated by entecavir. Hepatol Res 38: 842–846. 18498361 10.1111/j.1872-034X.2008.00332.x

[45] Cabuang LM, Shaw T, Littlejohn M, Colledge D, Sozzi V, et al (2012) In vitro replication phenotype of a novel (−1 G) hepatitis B virus variant associated with HIV co-infection. J Med Virol 84(8): 1166–1176. 22711344 10.1002/jmv.23328 PMC3538154

[46] Bock CT, Tillmann HL, Maschek HJ, Manns MP, Trautwein C (1997) A pre-S mutation isolated from a patient with chronic hepatitis B infection leads to virus retention and misassembly. Gastroenterology 113: 1976–1982. 9394738 10.1016/s0016-5085(97)70018-0

[47] Chen CH, Hung CH, Lee CM, Hu TH, Wang JH, et al (2007) Pre-S deletion and complex mutations of hepatitis B virus related to advanced liver disease in HBeAg-negative patients. Gastroenterology 133(5): 1466–1474. 17915220 10.1053/j.gastro.2007.09.002

[48] Kew MC, Kramvis A, Yu MC, Arakawa K, Hodkinson J (2005) Increased hepatocarcinogenic potential of hepatitis B virus genotype A in Bantu-speaking sub-saharan Africans. J Med Virol 75: 513–521. 15714494 10.1002/jmv.20311

[49] Hsieh YH, Su IJ, Wang HC, Chang WW, Lei HY, et al (2004) Pre-S mutant surface antigens in chronic hepatitis B virus infection induce oxidative stress and DNA damage. Carcinogenesis 25: 2023–2032. 15180947 10.1093/carcin/bgh207

[50] Märschenz S, Endres AS, Brinckmann A, Heise T, Kristiansen G, et al (2006) Functional analysis of complex hepatitis B virus variants associated with development of liver cirrhosis. Gastroenterology 131(3): 765–780. 16952546 10.1053/j.gastro.2006.07.008

[51] Chandra PK, Biswas A, Datta S, Banerjee A, Panigrahi R, et al (2009) Subgenotypes of hepatitis B virus genotype D (D1, D2, D3 and D5) in India: differential pattern of mutations, liver injury and occult HBV infection. J Viral Hepat 16(10): 749–756. 19457142 10.1111/j.1365-2893.2009.01129.x

[52] Soussan P, Pol S, Garreau F, Brechot C, Kremsdorf D (2001) Vaccination of chronic hepatitis B virus carriers with preS2/S envelope protein is not associated with the emergence of envelope escape mutants. J Gen Virol 82(Pt 2): 367–371. 10.1099/0022-1317-82-2-367 11161275

[53] Lee SA, Kim K, Kim H, Kim BJ (2012) Nucleotide change of codon 182 in the surface gene of hepatitis B virus genotype C leading to truncated surface protein is associated with progression of liver diseases. J Hepatol 56(1): 63–69. 21827734 10.1016/j.jhep.2011.06.028

[54] Mbarek H, Ochi H, Urabe Y, Kumar V, Kubo M, et al (2011) A genome-wide association study of chronic hepatitis B identified novel risk locus in a Japanese population. Hum Mol Genet 20(19): 3884–3892. 21750111 10.1093/hmg/ddr301

